# Alginate for cardiac regeneration: From seaweed to clinical trials

**DOI:** 10.21542/gcsp.2016.4

**Published:** 2016-03-31

**Authors:** Albert Liberski, Najma Latif, Christophe Raynaud, Christian Bollensdorff, Magdi Yacoub

**Affiliations:** Qatar Cardiovascular Research Center, Doha, Qatar

## Abstract

Heart failure is a growing endemic in the aging Western population with a prevalence of over 20 million people worldwide^1^. Existing heart failure therapies are unable to reverse heart failure and do not address its fundamental cause, the loss of cardiomyocytes^2^. In order to induce myocardial regeneration for the myocardium and the heart valve, facilitate self-repair, improve tissue salvage, reduce or reverse the adverse-remodeling and ultimately achieve long-term functional stabilization and improvement in the heart function, novel strategies for therapeutic regeneration are being developed which are aiming to compensate for the insufficient and low intrinsic regenerative ability of the adult heart^3^. Similarly, valve replacement with mechanical or biological substitutes meets numerous hurdles. New approaches using multicellular approaches and new material are extensively studied. Most of those strategies depend on biomaterials that help to achieve functional integrated vasculogenesis and myogenesis in the heart/tissue. Especially for failed heart valve function a number of therapeutic approaches are common from corrective intervention to complete replacement^4^. However the complexity of the heart valve tissue and its high physical exposure has led to a variety of approaches, however therapeutic regeneration needs to be established. Beside other approaches alginate has been identified as one building block to achieve therapeutic regeneration.

Alginate is a versatile and adaptable biomaterial that has found numerous biomedical applications which include wound healing, drug delivery and tissue engineering. Due to its biologically favorable properties including the ease of gelation and its biocompatibility, alginate-based hydrogels have been considered a particularly attractive material for the application in cardiac regeneration and valve replacement techniques. Here, we review current applications of alginate in cardiac regeneration as well as perspectives for the alginate-dependent, cardiac regeneration strategies.

## Background: General properties of alginate

**Figure 1.**
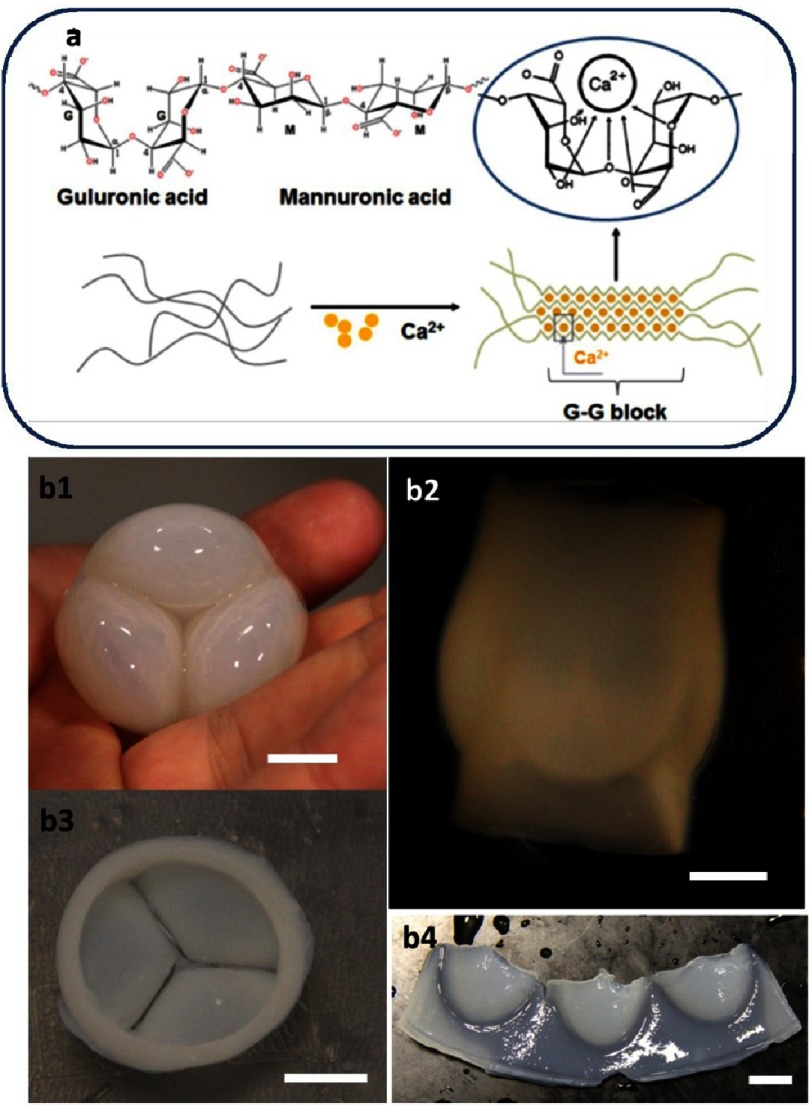
Alginate structure. The schematic model of hydrogel formation (A). Reprinted with permission from [2]. Versatility of alginate shaping. Alginate shaped in tricuspid valve, ventricular view (B1), atrial view, side view (B2), hinge - atrial view (B3), and open valve view (B4). (Scale bars 1 cm.)

Alginate is a naturally occurring polysaccharide typically extracted from brown seaweeds and has been widely utilized as a hydrogel for tissue engineering^5^. Alginate can be obtained with a wide range of molecular weights (typically 32–400 kDa)^5–7^ and is characterized by long chains that contain two different acidic components; namely, α-L-guluronic acid (G) and 1,4-linked β-D-mannuronic acid (M) (see Figure 1).

The overall ratio of both acids, M/G, and the way in which the G and M units are arranged in the chain, can vary from one species of seaweed to another. The origin and conditions of the extraction procedure can affect the viscosity of aqueous alginate solutions, lowering it if conditions are too arduous.

The strength of the alginate hydrogels can also vary from one alginate to another. For example, with a careful extraction procedure, *Laminaria hyperborea* gives strong hydrogels, while *Laminaria digitata* gives a soft-to-medium strength hydrogels^8^. Bacterial biosynthesis may provide an alginate with more defined chemical structures than that obtained from seaweed-derived alginate^9^. Generally, alginates with low M/G ratio will give a stronger hydrogels.

Alginates possess several crucial properties which make them suitable for use in cardiac regeneration. The first is their ability to be dissolved in water to yield aqueous solutions with moderate viscosity, which is particularly important for formulating injectable mixtures for cardiac therapies. The second is their ability to form hydrogels in mild conditions, for example by adding calcium salt to an aqueous solution of alginate. The calcium cations displace the sodium from the alginate, and grasp the long alginate molecules together, resulting in a hydrogels (see Figure 1). This property could be used to immobilize the cells in the desired area of the heart.

In contrast to the agar gels, where the solutions must be heated to about 60–80°C (depending on type) to dissolve the agar and the gel forms when cooled below approximately 40°C, no heat is required to form the alginate hydrogel; moreover, the hydrogels do not melt when heated. The ionically-triggered gelation of alginate is chemo-reversible, while covalent cross-linking is permanent (most often by standard carbodiimide chemistry or photo crosslinking^10^), and therefore, it is possible to modulate degradation rates and mechanical stiffness by choice of the appropriate crosslinking agents^11,12^. Different chemical and photo based ways can be used to do some crosslinking^10,13–17^.

The next pivotal property of alginates is their ability to form films^18,19^, fibers^20–22^, beads^23^ and virtually any shape in a variety of sizes (see Figure 1C). Moreover, as the partial oxidation of alginate does not significantly interfere with its hydrogels forming capability, the customized degradation of alginate could be induced by periodate oxidation of the alginate backbone^24^.

Within the field of biomedicine, the scope of alginate application is broad and includes wound healing, cell transplantation, delivery of bioactive agents such as chemical drugs and proteins, heat burns, acid reflux and weight control applications^5,25^. The non-thrombogenic nature of this polymer has made it an attractive candidate for cardiac applications^26–28^, including scaffold fabrication for heart valve tissue engineering^29^.

**Figure 2.**
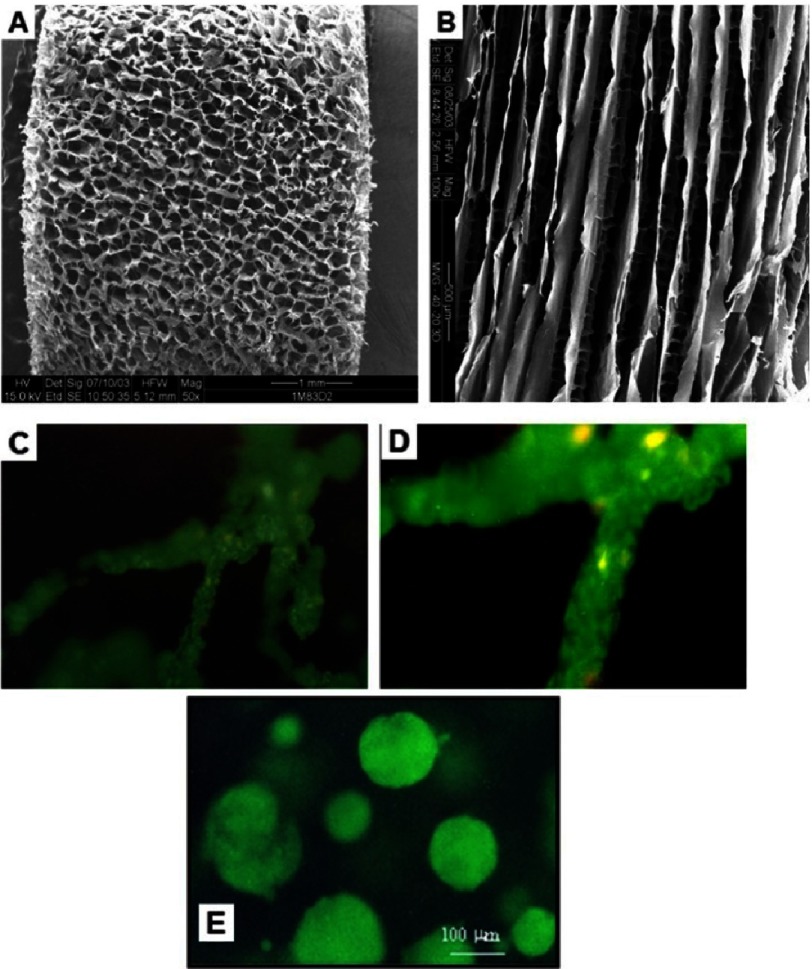
Alginate scaffold architecture affects tissue morphology and cell behavior. Depending on the freezing regime, the scaffolds can be prepared with isotropic (A) or anisotropic (B) pore structure. The pore dependent cell organization in the scaffold: cells cultivated in anisotropic (C, magnified in D), and spheroids grown in isotropic alginate scaffolds (E). Reprinted with permission from [32].

There are two main solid forms of alginate used in cardiac regeneration; namely, hydrogels and porous 3D scaffolds. The hydrogel can contain over 99% water trapped in the network of water-insoluble polymer chains. Two groups demonstrated that using a three step protocol where alginate is first cross-linked before being frozen and finally dried and this freezing method could dictate the organization of the pores created in the scaffold. If the freezing of the cross-linked alginate is done in a homogenous atmosphere of –20C the resulting scaffolds have an isotropic structure with spherical pore, while if freezing is performed by dipping one end of the scaffold in liquid nitrogen, this create a gradient of temperature leading to an anisotropic structure (see Figure 2)^30–32^. This technique was later commercially used by Life Technology inc. to propose a commercial 3D cell culture system with possibility of orientation under the name of AlgiMatrix™.

Depending on the freezing regime, the hydrogels could become 3D scaffold with interconnected pores (up to 200 µm in diameter, and 90% of matrix porosity)^32^. Ruvinov et al. reported that freezing of calcium cross-linked alginate solutions in homogenous cold atmosphere yields isotropic pore structure with interconnected spherical pores, while applying temperature gradient results in an anisotropic pore structure (see Figure 2)^32^. Importantly, the alginate structure architecture was shown to influence the cells’ organization (spheroidal, anisotropic) within the pores.

## Alginate contaminants and related hazards

There are several potentially harmful contaminants that may be carried by alginates and even accumulated during the alginate production processes. The most important from the point of treating heart failure are: heavy metals, formaldehyde, polyphenolic compounds and bacteria. In the case of alginates derived from seaweeds, the heavy metal content depends on the area and time of plant harvesting. Despite the strict regulation many ships are painted using paints containing heavy metals that prevent the growth of marine organisms^33^. The paints can release the metals which may accumulate especially in seaweeds that grow close to the sea communication routes. Moreover, despite strict regulation, some ships’ owners choose to cut the exploitation costs by disposing of the waste oils directly to sea water. This may also locally increase the amount of heavy metals in seaweeds exposed to such wastes. Heavy metals have a high affinity for sulfhydryl (-SH) groups, inactivating numerous enzymatic reactions, sulphur-containing antioxidants and amino acids with subsequent increased oxidative stress. This may result in vascular disease, hypertension, coronary heart disease and myocardial infarction^34^.

As with all polymers, alginates can also be degraded by reactive oxidate species. The formation of these species may be catalyzed by Cu^+2^, Fe^+2^ and Fe^+3^ ions. Those ions have a high affinity for alginates and can accumulate in their production. Therefore, these species should be removed from alginate, especially for its use in biomedical applications^35^.

Similarly aldehydes could be transferred from seaweed to the heart with an alginate injection, nevertheless animal studies showed that the direct action of formaldehyde on the heart plays only a minor role^36^. The negative chronotropic effect of formaldehyde in animals is caused mainly by the inhibition of sympathetic nervous activity through the central nervous system.

Polyphenolic compounds could be found in alginates. Surprisingly most of literature sources mention its positive effect on the heart mediated by reduction of oxidative stress^37^. Nevertheless, Paracelsus’ law states the action of a chemical depends on the dose of active agent. It is known that certain polyphenols may have genotoxic and carcinogenic effects. Furthermore, polyphenols may indirectly inhibit iron absorption which may lead to local iron depletion. Moreover, polyphenols may interact with certain pharmaceutical agents and enhance their biologic effects^38^. Nothing is known about administrating polyphenols directly to the heart, therefore removing polyphenolic compounds from biomedical alginates should eliminate possible risks.

Other potential contaminants of alginates include mould, yeast and bacteria, the latter may contribute to the presence of endotoxins, mitogens and pyrogens^35^. Considering that proinflammatory cytokines are now thought to play a key role in the pathophysiology of chronic heart failure^39^, this contaminant must be reduced, if not eliminated, from biomedical alginates.

Importantly, ultrapure alginates with strict control of alginate specification can be obtained *via* bacterial biosynthesis^40^. This enables controlling polymer molecular weight, its distribution, proportion and arrangement of G and M segments.

The production of biomedical alginates is strictly regulated by legislation enforced by a number of agencies. For example in USA the FDA is responsible for monitoring production of biomedical alginates^35^. Due to sharply defined requirements for composition and purity of biomedical alginate, the price of it is a hundred fold higher than alginates used in food industry. Therefore, to conduct early stage/ proof of concept experiments, the cheaper product may be used but only with awareness of restrictions that will require upgrading when approaching clinical applications.

## Alginate hydrogel biodegradation and clearance

The degradation, uptake and metabolism of alginate occurs in several types of bacteria. The class of enzymes called alginate lyases (Alys) catalyzes an endolytic β-elimination reaction that leads to depolymerization of alginate into oligomers^41^. Exolytic enzymes known as oligoalginate lyases are responsible for further degradation of oligomers into unsaturated monomers. Some specialized microorganisms degrade alginates and produce ethanol. This process could be efficient enough to consider alginate as feedstocks for bioconversion into biofuels and commodity chemical^42^.

Importantly, the same or similar degradation pathways were not discovered in mammals, thus alginate polymers are considered not to be biodegradable in the human organism^32^. One benefit resulting from that discrepancy between bacteria and mammals is that enzymes such as Alys can be safely used to remove alginate from maturing cell-laden scaffolds. This was reported by Takeuchi, who enzymatically removed the alginate envelope without harming encapsulated cells in the process of production of the “living threads”^21^.

In the literature there are frequently repeated opinions describing chains of alginate to be stable under physiological conditions^32,43,44^. Unfortunately such observations are insufficient to provide any insight into the fate of alginate injected in the heart. In this work, we present available reports retrospectively and our direct contribution into understanding what could happen with alginate injected into the human heart is therefore limited to identifying the niches and needs for further research. Below we are presenting several reports that can be used as a starting point for further experimental efforts.

Perhaps the most comprehensive, but still not gap- free discussion on alginate degradation, was proposed by Andersen and colleagues^35^. In their dissertation, they referred to the most commonly applied way of cross-linking the alginate with calcium ions. The first step to degradation in that case would be disintegration of material due to exchange of calcium ions by sodium ions in the physiological environment. The process of hydrogel disintegration and subsequently releasing alginate in its liquid form is the only way alginate from the hydrogel can leave the heart. The kinetics of this process is very difficult to anticipate and more experimental work is needed. It was clearly proven that even under physiological blood concentration of Ca^2+^ and Na^+^ ions, the alginate in a form of hydrogel can still subsist^45,46^, so it is reasonable to believe that alginate injected into the heart will be long lasting even after the concentration of Ca^2+^ in the hydrogel drops to the physiological level. The plasma sodium concentration^47^ is simply insufficient to completely hinder the cross linking effect of Ca^2+^^45,46^. Whenever the alginate polymer molecule finds its way into the blood, the following cascade of events occurs.

After hydrogel degradation the subsequent degradation of alginate will depend on the rate of cleavage of the glycosidic linkages in polymer chains. Anderson et al.^37^, estimated that alginate with Mw 200000 Da will degrade to 100000 Da at pH 7.4 and 37°C in 80 days. This renders the alginate as stable in physiological conditions.

The exact mechanism of alginate processing in the human body has not been yet elicited. The only light on that matter was illuminated by Duncan et al.^48^. To monitor the fate of alginate following intravenous, intraperitoneal and subcutaneous administration into mice, they developed a method based on the covalent incorporation of tyrosinamide into the alginate chains. The product could be radioiodinated to a high specific activity. Twenty-four hours after intravenous administration the larger polymer fraction remained in the circulation and did not readily accumulate in any of the tissues, while the low molecular weight fraction (≤48,000) of the injected polymer was excreted in the urine. At the same time point after intraperitoneal injection almost all dose of labeled alginate transferred from the peritoneal cavity to the blood compartment. Following subcutaneous administration, more than 70% of the injected dose was retained at the site of injection at 24h.

Even if the hydrogel decomposes, the average molecular weights of many commercially available alginates are higher than the renal clearance threshold of the kidneys. Therefore, the alginates should be carefully composed of a molecular weight, to enable controlled degradation and complete or close to complete removal from the body. To serve the proposed governing degradation rates, there are a few well-described tools. For example, at pH 7.4, alkaline β elimination is dominating the degradation path. This may be increased 5-fold by introducing to alginate chains the Periodate oxidate residues^35,49^. The side effect is to increase chain flexibility which leads to forming weaker gels. The degradation of alginates can also be enhanced by Gamma irradiation^50^. In that case, the degradation seems to follow the pattern of random depolymerization with no preference for specific residues^35^.

Many signaling pathways of cardiac cells, especially regulating heart performance^51^, depends on the concentration of calcium. The calcium injected into the heart with alginate hydrogel may cause long lasting environmental changes to surrounding tissue^45^, which may result in impaired myocardial function and cardiac relaxation and ultimately in heart failure^52^. Therefore, to avoid the risk, the amount of the calcium in the hydrogel should be close to physiological levels. In one report authors used 1% (wt/vol) alginate and 0.3% (wt/vol) calcium gluconate^53^ so the concentration of Ca^+2^ is about 28 mg in 100 mL of hydrogel, while normal blood calcium concentration is up to 10.5 mg per 100 mL. This makes the question of kinetics of release of calcium from alginate hydrogel even more vital.

## Current application of alginate as a biomaterial in cardiac regeneration

Currently the most advanced clinical trials, involving alginate based systems, for cardiac regeneration, includes Algisyl-LVR™ sponsored by LoneStar Heart, Inc (Laguna Hills, CA, USA) and PRESERVATION 1 sponsored by Bellerophon BCM LLC (Hampton, NJ, USA). These trials were preceded by extensive animal studies (pigs, dogs and rats), the key part of which is summarized below.

**Figure 3.**
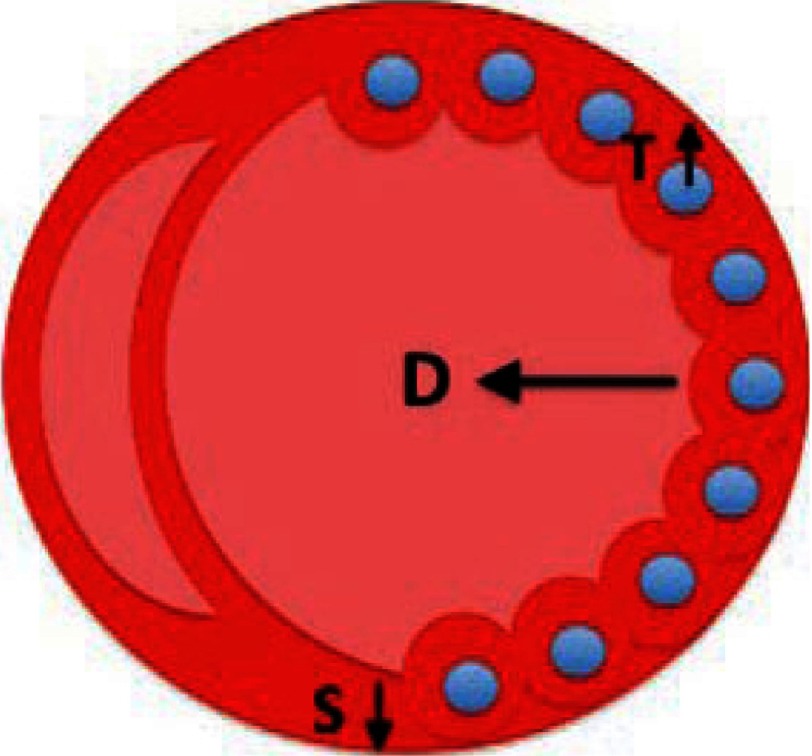
Schematic overview of restoration of the dilated ventricle. In accordance to Laplace’s law, left ventricular wall stress (S) is reduced by increasing the thickness of the wall (T), and reducing chamber diameter (D). This occurs due to the alginate injections; consequently progressive remodeling may be reversed or stabilized. Reprinted with permission from [56].

**Figure 4.**
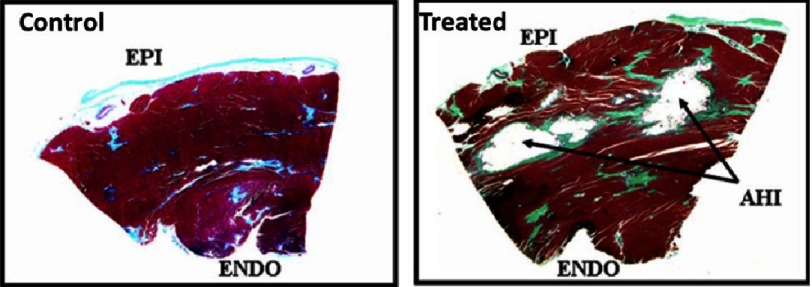
Transmural section from the LV in a control dog and a dog treated with alginate hydrogel implant (AHI). Reprinted with permission from [54].

###  Ventricle restoration by injecting cell-free alginate implants into the LV wall

The group of Randal J Lee evaluated the efficacy of alginate hydrogel implants in dogs with heart failure (HF) induced by repetitive coronary microembolization^54^. During an open-chest procedure, dogs received the injection mixture prepared by combining sodium-alginate aqueous solution with calcium cross-linked alginate hydrogel. Two weeks after the last microembolization, material was applied by 7 injections into the LV wall, halfway between the base and the apex, see Figure 3. After 24 h, the electrocardiography showed no significant differences between saline-treated and the alginate implant groups of animals with respect to severity of ventricular arrhythmias, heart frequency and rate. Four months post treatment the implant material was encapsulated by a thin layer of connective tissue still within the LV free wall. No evidence of inflammation was observed, see Figure 4. Compared to control, the alginate implantation significantly increased ejection fraction, wall thickness, improved LV sphericity, reduced LV end diastolic pressure as well as end-diastolic and end-systolic volumes.

These encouraging preclinical results have led to the initiation of clinical investigation for intramyocardial delivery of alginate implants in patients with acute myocardial infarct (MI).

Alginate implants, under the name of Algisyl^®^, were classified as a medical device (disputable, official FDA qualification), consist of Na^+^-alginate and Ca^2+^-alginate^55^, are dedicated for patients with enlarged left ventricle (LV) resulting from a mitral valve regurgitation, MI, arrhythmias and ischemia. The product comprises alginate that is administered directly into the left ventricle wall using up to 19 injections. Alginate remains in the heart muscle acting to increase pumping efficiency, reduce left ventricular wall stress, and prevent further dilation and negative remodeling of the left ventricle (see Figure 3).

Also in human studies^56^, implementing alginate within the ventricular wall has been shown to reduce the wall stress of the dilated heart and relieve the muscle tension^56^. The studies by Lee et al. indicated significant improvements in the ejection fraction (18% increase after 3 months post injection) and other cardiomechanical parameters, all summarized in Table 1 and favourable biological effects such as tissue regeneration and neovascularization.

**Table 1.** In-man experience with LV restoration in patients with systolic heart failure (reprinted with permission from [54] ).

	Presurgery	Post 3 days	Post 8 days	Post 3 months
LVEF (%)	28.7 ± 8.5	37.6 ± 11.2	36.5 ± 16.0	36.0 ± 13.5
LVEDV (ml)	139.5 ± 20.6	122.5 ± 13.9	123.5 ± 45.0	123.6 ± 18.6
LVESV (ml)	99.8 ± 25.8	79.5 ± 22.8	87.2 ± 46.0	77.2 ± 29.5
KCCQ score	39.4 ± 28.0	n/a	53.4 ± 19.9	74.0 ± 25.0*
No. of patients in NYHA class III/IV	6	n/a	1	1

**Notes.**

This is a synopsis of the data presented at the 2010 American Heart Association (AHA) meeting by Dr. Bauernschmitt’s group (^∗^*p* < 0.05). LVEF, left ventricular ejection fraction; LVEDV, left ventricular end diastolic volume; LVESV, left ventricular end systolic volume; KCCQ, Kansas City Cardiomyopathy Questionnaire; NYHA, New York Heart Association.

The other advantages of this alginate based treatment are that it does not interfere with drug therapies and procedures such as valve or bypass surgery^57^, transplantation or Left Ventricular Assist Devices (LVADs). Moreover, as a one-time procedure, it is less time consuming, more efficient and cost-effective in comparison to active implantable devices that require a power source. Since the group of patients in the Lee studies was small, namely two patients with non-ischemic and four patients with ischemic dilated cardiomyopathy, and follow-up of 3 months only on one patient, the results were to be confirmed in clinical trials on a wider group of 76 patients (ClinicalTrials.gov Identifier: NCT01311791). Final data collection date for primary outcome measure was assigned on April 2014, but the estimated study completion date is April 2016. This study is ongoing and no results currently posted on ClinicalTrials.gov.

Subsequently, the manufacturer of Algisyl-LVR (LoneStar Heart, USA), instigated a research grant with the objective of comparing alginate-hydrogel injections with standard medical therapy to determine the impact on clinical outcomes in patients with advanced heart failure^58,59^. The authors found that alginate-hydrogel injections in addition to standard medical therapy for patients with advanced chronic HF was more effective than standard medical therapy alone for improving symptoms and exercise capacity. Thirty-five patients were treated with thoracotomy assisted, alginate-hydrogel injections^59^. Alginate-hydrogel treatment was associated with improved peak oxygen uptake on cardiopulmonary exercise^59^ at 6 months compared to control. Also six-minute-walk-test distance improved in alginate-hydrogel-treated patients versus patient treated by standard medical therapy. The study was conducted at 14 centres in Australia, Germany, Italy, the Romania, and Netherlands, however researchers should have indicated more precisely what “standard medical therapy” means, as it may be an important variable. Unfortunately, the 30-day surgical mortality was 8.6% (3 deaths) which is not superior but neither inferior in comparison to the outcome of other devices currently under investigation, for example HeartNet device^60^ (Paracor Medical Inc.), the Parachute device^61^ (Cardikinetix), Revivent device^62^ (Bioventrix).”

## Treating myocardial infarction by injecting alginate in coronary artery

**Figure 5.**
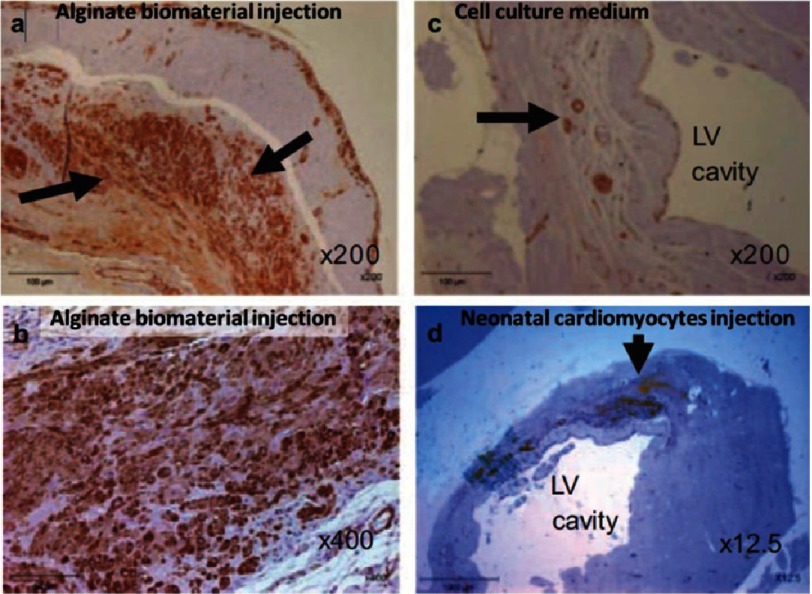
Infarcted rat hearts after immunostaining for α-SMA, after treatment with alginate or cardiomyocyte transplantation. In the scar tissue 8 weeks after alginate injection, extensive positive brown staining revealed that the scar was populated with α-SMA positive myofibroblasts (A,B). Fewer myofibroblasts were observed for control scar to which the cell culture medium was injected in place of alginate (C). Neonatal cardiac cell implant (arrow) at the border of the infarct zone 8 weeks after transplantation (D). Engrafted cells were undifferentiated and isolated from the host myocardium. Reprinted with permission from [53].

Similar way of applying alginate to cardiac regeneration was investigated by Landa et al.^53^. In this study rats were used as a model. Calcium-crosslinked alginate solution was prepared from an aqueous solution by mixing it with calcium gluconate solution. Importantly, for temporal tracking of the injectable alginate biomaterial in infarcted hearts, biotin-labeled alginate was used. This was injected into the infracted area 7 days after anterior myocardial infarction. *In situ* formed hydrogel occupied 50% of the scar area. Alginate was replaced by connective tissue within 2 months, echocardiography studies showed that injection of alginate biomaterial reduced left ventricular diastolic and systolic dilatation, and dysfunction. The results were compared to those achieved by neonatal cardiomyocyte transplantation and were found to be superior (see Figure 5).

Authors concluded that injection of bioabsorbable, *in situ*–forming, alginate hydrogel is a feasible acellular strategy that prevents adverse cardiac remodeling and dysfunction in recent and old myocardial infarctions. Authors avoided addressing the potential risk of calcification of heart tissue related to the increased concentration of calcium ions. Depending on the method a 5% CaCl_2_ (∼ 450mM) solution can be used to harvest solidified alginate^63^. Perhaps this is due to recent reports describing anti-mineralization effect of alginate on porcine and human cardiac valves^64^.

**Figure 6.**
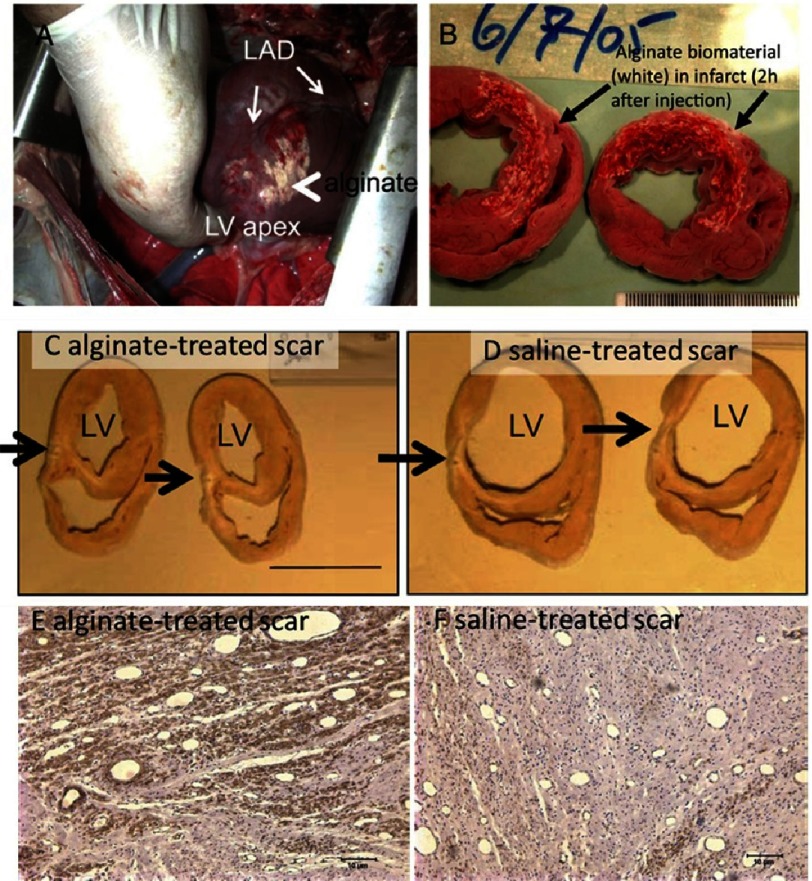
Infarcted heart after intracoronary injection of calcium cross-linked alginate solution. 120 min after injection, the alginate hydrogel was identified on the infarct surface (A) (arrows). Heart sections revealed hemorrhagic infarct and in situ deposition of the alginate hydrogel (white areas, arrows) and consistent distribution of the biomaterial in the granulation tissue (B). Increased thickness of the scar (black arrows) was induced by alginate implant 60 days after (C & D). Infarcted hearts after immunostaining for α-SMA, 3 months after alginate (E) or saline injection (F). Smooth muscle actin positive myofibroblasts were predominantly observed when alginate was injected to scar area. Reprinted with permission from [65].

Similar animal studies were carried out on larger animals by Leor and colleagues^65^. They prepared calcium cross-linked alginate solution that undergoes liquid to hydrogel phase transition as previously studied after injection into the infarct-related coronary artery in swine. Examination of hearts harvested 2 hours after injection showed that the alginate crossed the infarcted leaky vessels and was deposited as hydrogel in the infarcted tissue (see Figure 6A and B).

The authors clearly establish the increased number of myofibroblasts over fibroblasts in alginate treated tissues compared to saline treated ones. This increased number of myofibroblasts is believed to be of better outcomes for multiples reasons. First, it is now established that fibroblasts are involved in formation of the extracellular matrix and the extent of the extracellular matrix. Excess collagen deposition with a loss of collagen degradation by matrix metalloproteinases leads to fibrosis which has a long term impact on the function of the heart. Separation of myocytes by collagenous septa insulates myocytes and decreases myocyte-myocyte interactions leading to an overall loss of normal function. Those fibroblast can differentiate into myofibroblasts which are cells involved in the inflammatory response to injury, as they produce cytokines thus enhancing the inflammatory response. The complex intracellular structure of myofibroblasts define them with a contractile mechanism that enables these cells to generate force to the surrounding extracellular matrix. This contractile force is maintained over time and reinforced by the deposition of collagen.

Moreover, histological analysis performed 60 days after injection, showed that applying 2 mL of alginate solution increased scar thickness by 53% compared with control and was replaced by collagen and myofibroblasts (see Figure 6C-F). It highlights indirect improvement due to additional cells growing. At the same time point the control swine injected with saline solution experienced an increase in left ventricular (LV) diastolic area by 44% and LV systolic area by 45%. The authors concluded that intracoronary injection of alginate biomaterial is safe, feasible, effective, could be used as an intervention to prevent adverse remodeling and improve infarct repair after myocardial infraction. Unfortunately, the authors did not discuss risks related to increased calcium concentration induced by calcium-cross-linked alginate, which includes calcification of the heart tissues^64,66,67^.

Unlike in the previous approach where alginate is injected in the wall of the ventricle and remains in place encapsulated by a thin layer of connective tissue, when injected in the coronary artery, the alginate is rapidly replaced by collagen and myofibroblasts.

A very elegant, but still proof-demanding explanation, on how alginate acts in the infarct area was proposed by Ruvinov and colleagues in their outstanding review^32^. They concluded that the elevated calcium concentration at the acute infarct site causes local crosslinking of alginate, its rapid gelation and phase transition into a hydrogel. While the tissue is healing, the calcium concentration drops and alginate crosslinking reverse. Indeed, the average calcium concentration in the blood stream is 9.5 mg/dl, which is too low to maintain the grasp of the long alginate molecules together. After administration, the alginate hydrogel degrades and disappears from the infarct zone and is replaced by host tissue composed of myofibroblasts enriched with blood capillaries^53,65^.

These promising results have led to the launching of clinical investigations of intracoronary delivery of alginate biomaterial in patients with acute MI by Bellerophon (ClinicalTrials.gov Identifier: NCT01226563). They used the aqueous mixture of sodium alginate and calcium gluconate as a bioabsorbable cardiac matrix for preventing negative ventricular remodeling following acute myocardial infarction^68^. During standard catheterization procedure, the hydrogel is injected via the coronary artery into the damaged heart muscle. Here injected material perfuses and polymerises in sites in between the cells and fibers of damaged tissue. This provides physical support of the heart muscle during repair and recovery. It is claimed that alginate gradually dissipates and is excreted through the kidneys after healing^69^. While the author concluded on the safety and efficacy of this approach in human patients, no condition improvement was reported. Ongoing clinical trials involve approximately 300 patients who had successful percutaneous coronary intervention following acute ST-segment (ST-specific part of echocardiogram) elevation myocardial infarction (ClinicalTrials.gov Identifier: NCT01226563).

## Alginate for angiogenesis: controlled delivery and composites

The potential use of the different alginate hydrogels as pharmaceutical excipients has not yet been fully evaluated but alginate is likely to make an important contribution in the development of polymeric delivery systems. The precise chemical degradation mechanism and approaches in the choice of alginate for drug deliveries was previously broadly reviewed^70^. Briefly, the possibility to form two types of gel with alginate dependent on pH (acid gel) or ions, gives the polymer more tailored capacities compared to neutral macromolecules. More than 200 different alginate grades and alginate salts are now offered. When considered for local injection or oral delivery (oral route being the patient preferred drug administration), the type of hydrogel used is obviously crucial. Alginates polymers are one of the most extensively explored mucoadhesive biomaterials thanks to very good cytocompatibility and biocompatibility, biodegradation and sol-gel transition properties. But beyond the chemical composition and re-swelling approach chosen, multiple mechanisms can be considered to control the release speed of a compound using alginate. First, the size of the hydrogel can be controlled; a higher surface area will provide more “space” for the molecule to diffuse through the hydrogel. The permeability of the hydrogel is obviously critical as is the density of the polymer that controls it which can control the how easily the molecules can diffuse through the polymer network.

**Figure 7.**
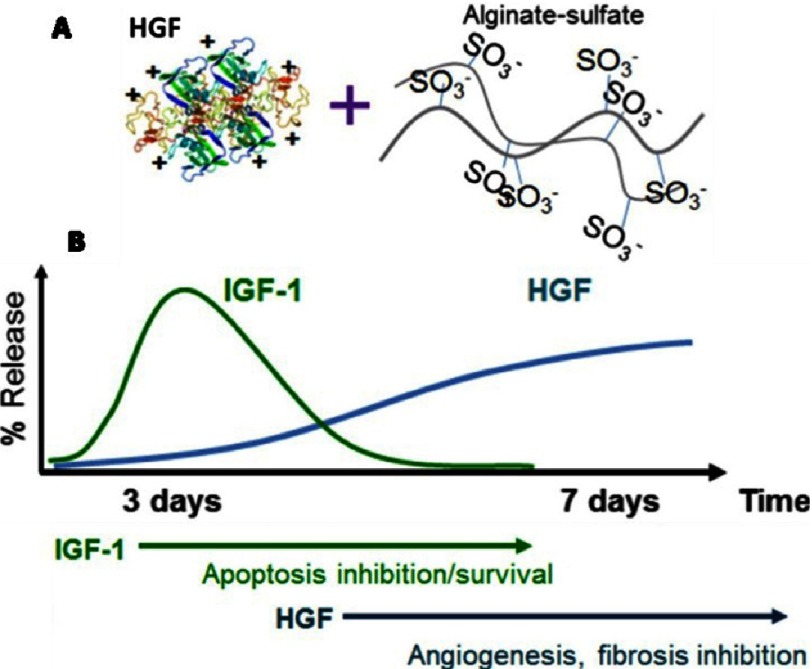
Schematic representation of the “anchoring” of the growth factor into alginate sulfate (A) and summary of sequential delivery of growth factors from alginate in vivo (B). The faster release of insulin-like growth factor 1 (IGF-1) could provide strong pro-survival signals at early stages, while the slower release of HGF could induce angiogenesis at later stages. Reprinted with permission from [75] and from [32].

Several laboratories have demonstrated the utility of alginate for the controlled delivery of angiogenic factors. The loss of ion in the alginate hydrogel can trigger their dissolution therefore crosslinking could be utilized to improve the mechanical stability and control the degradation kinetics of an alginate hydrogel^71–73^. For example, Vascular endothelial growth factor (VEGF) entrapped in the alginate hydrogel has a steady and continuous release for more than one month^74^. Free VEGF is degraded within 72 h post-injection while VEGF injected within the alginate hydrogel is detectable up to 15 days after injection. Moreover, to enhance affinity to angiogenic factors, alginate can be sulfated such that it mimics the structure of heparin^75^ (see Figure 7). This modification of the hydrogel has stronger binding to Hepatocyte growth factor (HGF) and achieved a higher therapeutic effect than the unmodified alginate. All this together makes alginate an interesting candidate as a biomaterial for enhancing angiogenesis in heart failure.

Hao and colleagues investigated if local sequential delivery of vascular endothelial growth factor (VEGF) followed by platelet-derived growth factor-BB (PDGF-BB) with alginate hydrogels could induce a functional improvement and angiogenic effect which is greater than single factors after myocardial infarction in a rat model^76^. Hydrogels were administered intra-myocardially along the border of the myocardial infarction. Hydrogels comprised low and high molecular weight alginate to enable control of the release kinetics of incorporated factors^74^. Hydrogels were capable of delivering the growth factors sequentially; the VEGF was released faster than PDGF-BB. This led to a higher density of α-smooth muscle actin positive vessels than in the case of single factors but no increase was found in capillary density. Sequential protein delivery increased the systolic velocity-time integral and displayed a superior effect than single factors. In the aortic ring model, sequential delivery led to a higher angiogenic effect than single factor administration^76^. Authors concluded that inducing mature vessels and improving cardiac function could be enhanced by applying the growth factors sequentially rather than individually indicating clinical utility.

Finally, alginate has been blended with a conductive polymer namely, polypyrrole to enhance cardiac cells–implant interaction. The alginate polymer blend was injected into the infarct zone of the rat heart. At 5 weeks post-treatment, the blend yielded significantly higher levels of arteriogenesis when compared with the alginate only treatment group and the saline control group. In addition, the blend enhanced infiltration of myofibroblasts into the infarct area^77^. The results of this study highlight the potential of combining alginate with other biopolymers for application in cardiac regeneration strategies.

In fact, all efforts aiming to enhance and control the conductivity of alginate based systems are substantial^77,78^. Electrical, signal may be transduced with different rate, around the alginate island; this may cause re-entry circuit and *in fine* arrhythmia^79^. Problem of that nature occurred when ischemic heart failure was treated by skeletal myoblast transplantation^80^. The one way of solving that problem would be to implant cardio-defibrillator (ICD), which can send a high-energy electrical signal to eliminate arrhythmias. Unfortunately, this device causes a highly unpleasant sensation felt by the patient^81^. In a different study it was shown that arrhythmia was avoided when undifferentiated human ESCs surgically delivered onto the infarct area in a 68-year-old patient were embedded into a fibrin scaffold^82^. This is encouraging evidence indicating the importance of applying hydrogel to enhance performance of cell-base therapies.

## Alginate based patches for cell transfer in cardiac regeneration

**Figure 8.**
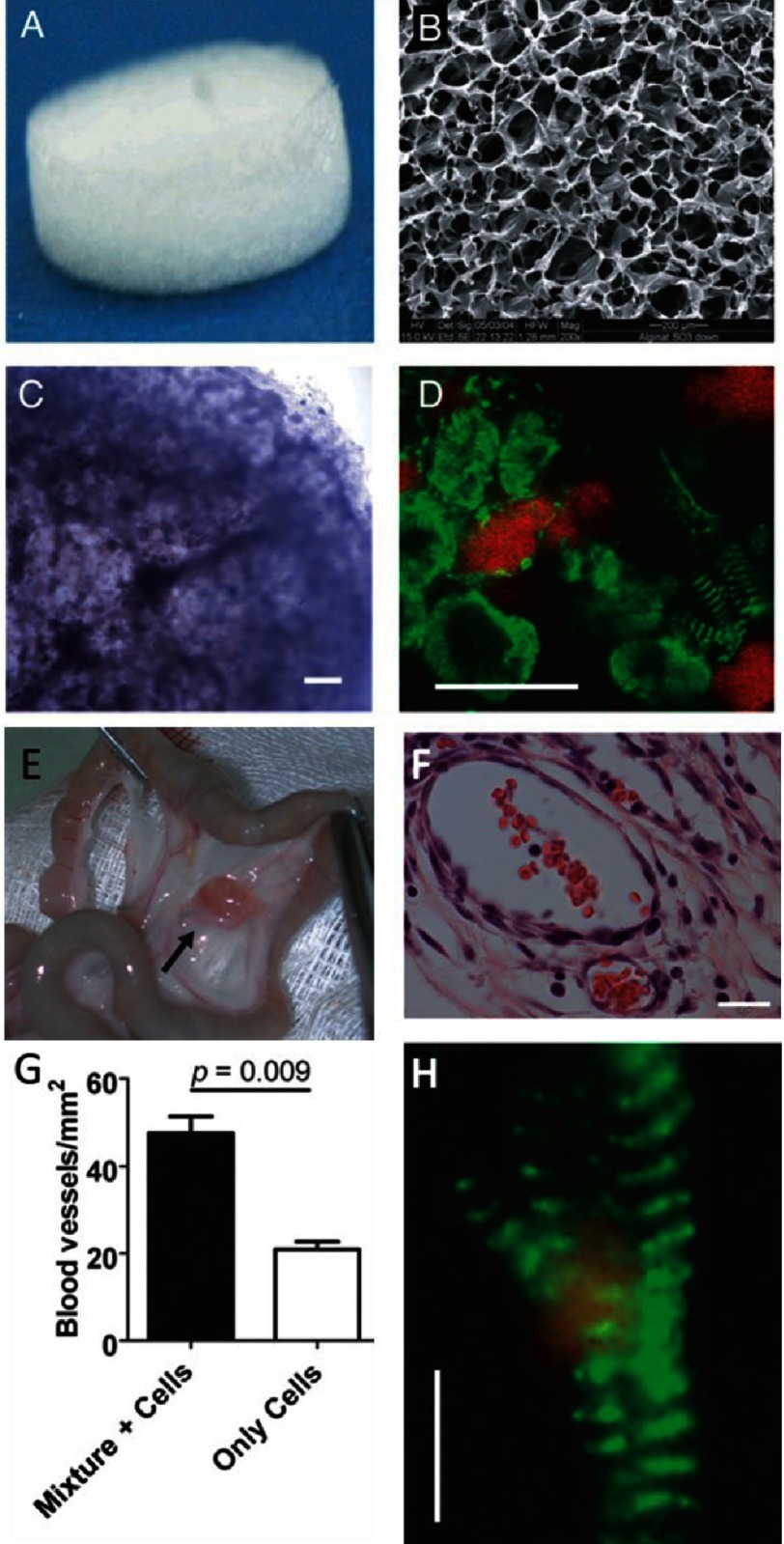
Construction of a cardiac patch in an alginate/alginate-sulfate scaffold capable of binding and releasing mixed factors. The scaffold features before cell seeding; macroscopic view (A) and internal porosity by scanning electron microscopy (B). Cardiac patch seeded with cells and supplemented with factors after 48 h of cultivation. Light microscope view of the cardiac patch showing uniform distribution of cells in the matrix pores (C). Cardiac cell organization within the scaffold as judged by anti-actinin immunostaining (green) and nuclear staining (red) (D). Some of the cells reveal the typical striation of cardiac tissue. Vascularization of the 7 day omentum-transplanted mixture supplemented cardiac patch. The cardiac patch (arrow) is stitched to the omentum (E). Mature blood vessels populate the cardiac patch supplemented with factor as judged by anti-SMA immunostaining (brown) (F). Blood vessel density in the omentum-implanted patches (G). Typical cardiac cell striation is revealed in an omentum generated, factor-supplemented alginate cardiac patch as revealed by anti-actinin immunostaining (green) and confocal microscopy (H). Scale bar: 200 µm (C&E); 10 µm (D&H). Reprinted with permission from [87].

It is evident that alginate mediated cell delivery can improve cellular viability and retention^65,83,84^. Building on this advantage, Dvir and colleagues^85^ constructed a patch by seeding neonatal rat cardiac cells isolated from the LV of 2 days old rat heart in Matrigel (authors failed to describe manufacturer of Matrigel) supplemented with a mixture of angiogenic and prosurvival factors (insulin-like growth factor-1 (IGF-1), stromal-cell derived factor 1 (SDF-1), and VEGF into macroporous alginate scaffolds. To promote maturation of vasculature the patch was cultured for a week in the rat omentum and transplanted into post-infarction rats. One month after implantation the patch showed electrical and structural integration with the host myocardium (see Figure 8).

**Figure 9.**
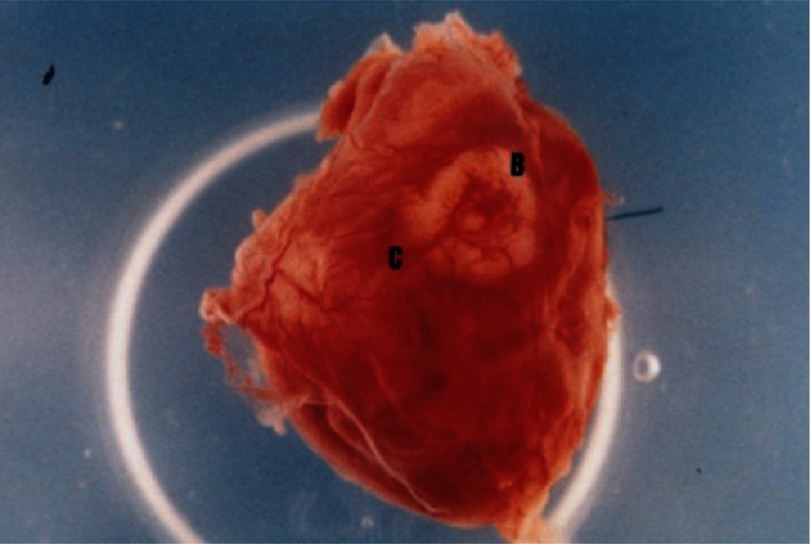
Photograph of heart at day 63 after implantation of cellular construct revealed intense neovascularization growth into biograft (B). Coronary branch (C) supplies biograft and covers it with network of vessels. Reprinted with permission from [28].

Similar results were reported by Leor and colleagues^28^ who successfully implanted cardiac cell-seeded macroporous alginate scaffolds into infarcted rat hearts^28^. After 24 h, the fetal rat cardiomyocytes seeded within the scaffolds formed multicellular beating cell clusters^27^. Following implantation of the construct with viable cells into the infarcted myocardium, some of those cells differentiated into mature myocardial fibers. Moreover, the graft itself and its surrounding tissue vascularized (see Figure 9).

**Figure 10.**
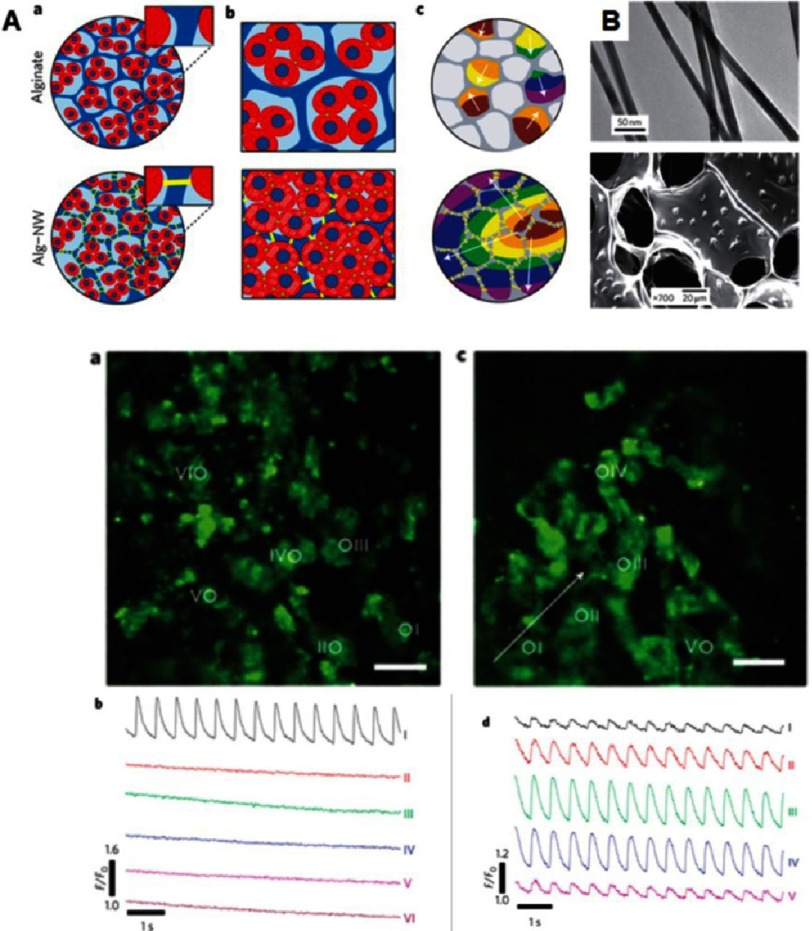
Cardiac tissue engineering in nano-composite multifunctional alginate scaffolds. Schematic overview of three-dimensional engineered nano-wired cardiac tissue: alginate pore walls (blue), cardiac cells (red) and gold nanowires (yellow) (A). Incorporation of nanowires within alginate scaffolds (B). Top: typical gold nanowires, bottom: nanowires assembled within the pore walls of the scaffold. Cardiomyocytes cultured in pristine alginate (a) and in alginate nanowires–composites (c). Monitoring calcium dye fluorescence for measuring calcium transient propagation within engineered tissues assessed at points marked with white circles. Calcium transients were only observed at the stimulation point in the unmodified scaffold (b). Calcium transients were observed at all points of wired composite (d). Reprinted with permission from [78].

The versatility of alginate enables the preparation of scaffold which is designed for a specific action and need. For instance Dvir et al. developed 3D nanocomposites incorporated gold nanowires within macroporous alginate scaffolds to bridge between the non-conducting pore walls. This enhanced the organization of functioning cardiac tissue by increasing electrical signal propagation throughout the cell seeded scaffold^78^ (see Figure 10). While addressing the risk of reduced propagation of the electric signal within the scaffold, additional work should be done to analyze the opposite risk that represents increased velocity of the electric signal propagation.

## Alginate derivatives for cell transfer in cardiac regeneration

**Figure 11.**
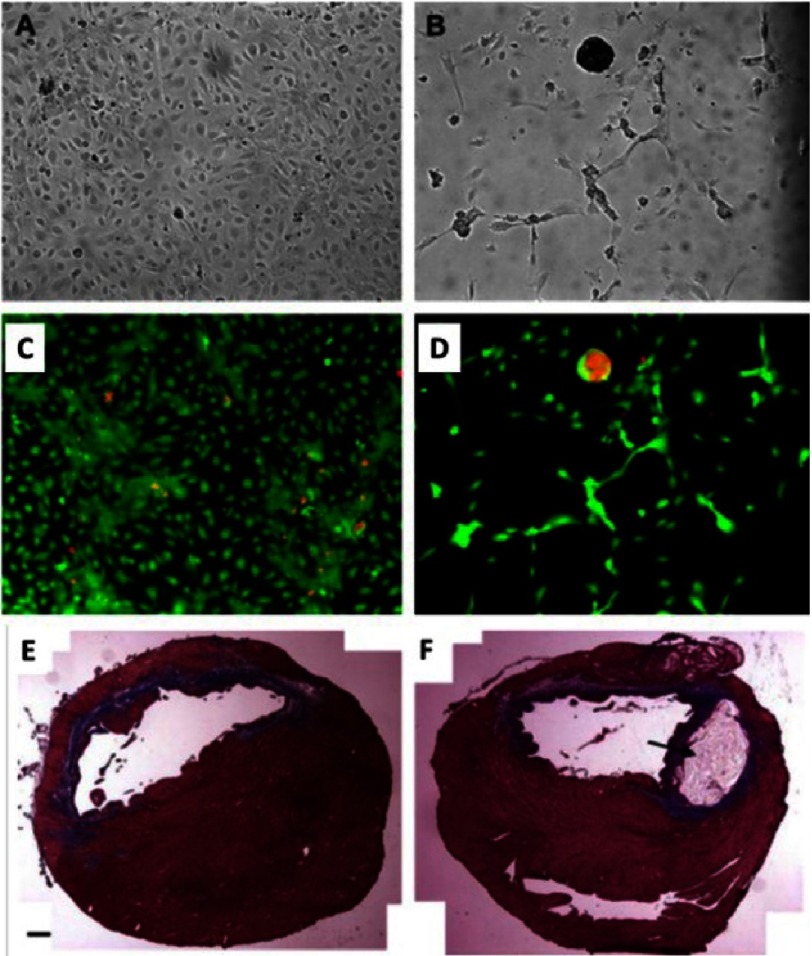
Culture of HUVECs after 5 days on RGD modified alginate (A) and non-modified alginate (B). Live (green)/dead (red) assay of (A) (C). Live (green)/dead (red) assay of (B) (D). Trichrome staining at 5 weeks post-injection of PBS (E) and alginate injected heart (F). Black arrow in (B) indicates residual alginate *in situ*. Scale bar: 1 mm. Reprinted with permission from [86].

To enhance cellular growth, alginate was modified with Arg-Gly-Asp peptide (RGD) (see Figure 11A–D). While injected up to 5 weeks post-MI in rat, either modified and unmodified alginate hydrogels led to increased arteriole presence, improvement in end diastolic and LV end systolic diameter compared with saline (see Figure 11E & F)^86^.

**Figure 12.**
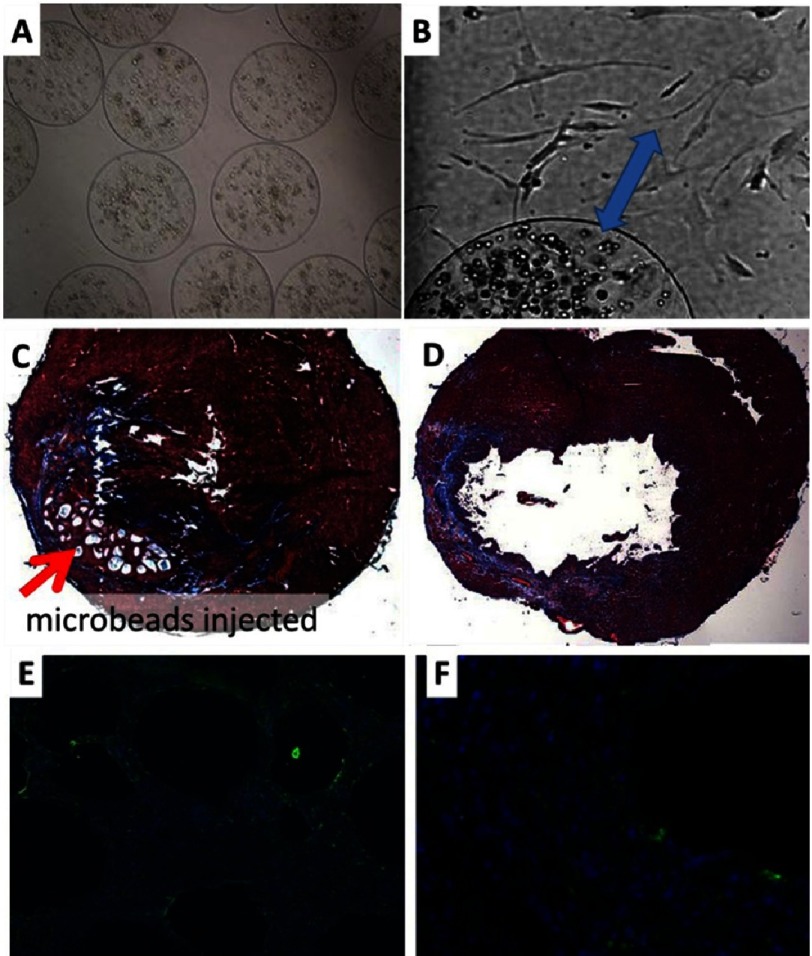
RGD-Alginate microbeads after human MSCs encapsulation. 2 days (A) and 5 weeks after encapsulation (B). Note: cell transfer to tissue culture plastic (blue arrow). 4x and 10x respectively. Trichrome staining of heart at 10 weeks post-injection of hMSCs encapsulated in RGD-Alginate, red arrow indicates beads (C). Injection of PBS (control) (D). Vimentin stain (green) for cell retention after 2 weeks post injection of hMSCs microbeads, (E) 10x, and (F) 40x. Blue color: nuclei stain. Reprinted with permission from [88].

Often the peptide-cell interaction could be specific to a given type of cell^89^; nevertheless, RGD-peptide -modified-alginate is versatile, thus it was also used to encapsulate human Mesenchymal Stem Cells (MSCs) for the transfer (see Figure 12. Noteworthy, *in vitro*, the cells in the beads after 5 weeks of incubation are still spherical. As it was shown before, in this shape, they are under the stress but can still grow and proliferate, nevertheless, in contact with a more cell-“friendly” surface, the cells migrate vigorously to it (see Figure 12B blue arrow). This cell affinity difference between the surfaces is vital for cell transfer applications. Injection of RGD-modified alginate microbeads without and with MSCs in a 1-week rodent MI, led to improvement in preservation of wall thickness, LV internal diameter and fractional shortening (FS)^88^. Nevertheless, the role and benefit of MSCs injection following MI remain controversial particularly in clinical applications^89^

Importantly, the experiment performed by Tsur-Gang et al.^90^ also on rats and in the similar time range, showed that unmodified alginate is superior in improvement of scar thickness and left ventricle dilatation and function, compared with alginates modified with RGD and YIGSR, as well as with RGE peptide. This discrepancy may be due to reduced coverage of the infarct resulting from alteration in viscosity of hydrogels upon covalent attachment of the peptides^32^. It must also be mentioned that covalently unbounded peptides entrapped in alginate may hinder the cellular affinity triggered by peptides covalently linked to alginate^91^. This is because free standing peptide when anchored to cell receptor blocks the cellular capacity for adherence. For this reason experimenter must take extra care for the purification of the product prior to using it. This simple validation of hydrogel purity was not presented in papers discussed here.

## Potential disadvantages of alginate and its alternatives

Despite its benefits, the alginate based hydrogels have a few important drawbacks. For example as a natural product its composition may vary from batch to batch which includes ratio of G- and M- building blocks, molecular weight and molecular weight distribution. This may have tremendous effect on gelation properties and mechanical properties of hydrogels which is pivotal for biopolymer performance as a treatment agent. Despite best efforts, it is still not fully understood. What is the final fate of the injected material? How it is eroded from the tissue and is excreted from the body? Moreover, in its cross-linked form alginate is a source of calcium ions. As discussed, that may be associated with a higher calcification risk. The vital question of whether the presence of calcium alginate in the human heart enhances, prevents or has no effect on tissue calcification, has not yet been fully addressed. That is mainly because the process of calcification and its background is complex and enigmatic, despite outstanding research on the topic^92^.

Alginate calcification has been reported during animal implantation^67,93,94^ and clinically^95^. Perhaps the most comprehensive studies on alginate mediated calcification and characterization of the mineral was provided by the Schwartz group^67^. The calcification process was investigated in nude mice by injecting subcutaneously or intramuscularly calcium-crosslinked alginate microbeads. In both cases the beads mineralized maximally 6 months after injection. In-depth analysis revealed that calcium phosphate deposited uniformly on the microbeads. The minerals on the beads’ surface closely matched hydroxyapatite found in bone. In the same study, the authors evaluated several methods to regulate or eliminate mineralization. They concluded that replacing calcium by barium, as well as buffering the crosslinking solution with HEPES, prevents calcification *in vivo*. This study confirmed that alginate processing prior to its implantation is necessary to regulate alginate calcification.

A potential way to circumvent the problem lies in a strategy of mixing the calcium-crosslinked alginate with an anti-calcification agent^57,68^. For example, sodium alginate was shown to slow down calcification^64^. Kanakis and colleagues crystalized calcium carbonate, using porcine and human cardiac valve leaflets as a substrate, with and without sodium alginate. For both human and animal tissues, addition of sodium alginate reduced the rates of crystal growth by over 40%.

It is reasonable to believe that calcification of tissue is regulated by many overlapping factors that optimally are in balance^92,96,97^. Indeed, calcium alginate can affect this balance severely by the adsorption and subsequent blocking of the active growth sites^98,99^. Nevertheless, the fact that in the clinical trials discussed above^57,68^ alginate was not associated with extensive tissue calcification points to its advantage as a therapeutic agent, although more research is needed on this subject.

Due to the potential drawbacks listed above, it is vital to compare this material to other alternatives that may be superior in any given application. For example, Zafar and coworkers recently reported bioscaffolds prepared from decellularised, non-crosslinked extracellular matrix (ECM) derived from small intestinal submucosa. It was used to prepare a tubular tricuspid valve that showed physiological growth, remodeling and preserved function^100^.

**Figure 13.**
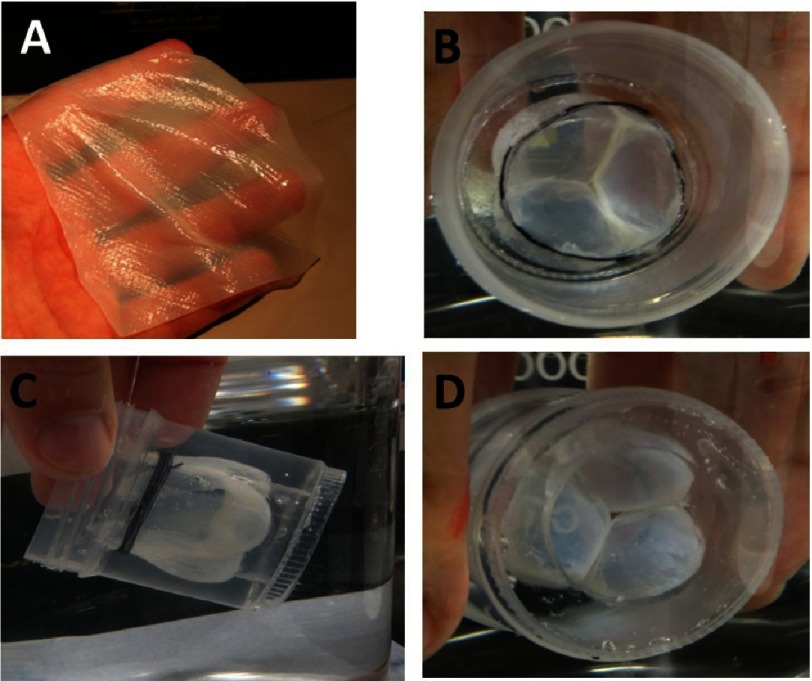
Bacterial nanocellulose in form of sheet 10×10 cm (A), leaflets immobilized in transparent PDMS (B-D). Hyaluronic acid-loaded nanocellulose sheets were provided as a gift by Bowil Biotech, Władysławowo, Poland.

Another potentially useful material for a variety of heart repair strategies is bacterial nanocellulose^101^. Its superior mechanical properties make it an extremely interesting candidate to test as a base for cardiac patches or to produce heart valve leaflets, see Figure 13 for unpublished data.

Also, chitin and chitosan (in combination with other biomaterials) were successfully used to produce scaffold for heart leaflets^102^. For the full range of physiological flow conditions tested, the electrospun gelatin-chitosan polyurethane demonstrated good biocompatibility and cell retention properties^103^.

Synthetic polymers may also potentially be used in therapies related to heart failure and tissue engineering^104^. For example elastomeric poly(glycerol sebacate) (PGS) was reported to support *in vivo* remodeling of cell-free synthetic graft into a neoartery^105,106^. Sohier and colleagues proposed applying nano-fibrillar anisotropic poly-(ε-caprolactone) (PCL), as a substrate for heart valve engineering^107^.

In their outstanding report Baaijens and coworkers claimed using poly-ε-caprolactone bisurea strips to modulate macrophage polarization within scaffolds and trigger specific immune response to enhance deposition of sulphated glycosaminoglycans and collagen on the scaffolds to support tissue remodeling^108^.

## Conclusions and future alternatives of alginate-based cardiac regeneration techniques

Thanks to a variety of crosslinking approaches, alginate can form versatile and tunable hydrogels, suitable for carrying cells and developing 3-D cellular microenvironments. Alginate can be modified with a variety of biological agents to promote matrix-cell interactions, guide 3-D cell organization, modulate cell fate and promote cellular integration^32^.

Alginate-based biomaterials for the treatment of MI is entering the clinical trials stage, therefore understanding the mechanisms by which these therapies affect LV remodeling, cardiac function and cardiac electrophysiology becomes a pivotal issue. Despite progress made in cardiac patches and injectable alginates, much of its potential remains unexplored, including alginate-mediated reprogramming of non-cardiomyocytes into cardiomyocytes^109^, alginate as a vehicle for delivering cardiac progenitors cells and their derivatives, alginate-supported generation of the new cardiomyocytes, and alginate-assisted activation of endogenous cardiac progenitor cells.

To fully utilize alginate in those applications several challenges still need to be addressed. Firstly, more cell-interactive alginate derivatives are required to offer a better precision in controlling cell organization, differentiation, growth and function. Governing at the micro- and nano-scale, the spatial distribution of signaling and topographical cues are key tools for designing cell-interactive systems. Next, more tunable alginate-based release systems need to be developed to improve their safety, obtain local effects and increase sustainability. The focal point of these efforts is the sequential release of drugs (small molecules, proteins, nucleic acids) while on-demand drug release could take place in response to signals derived from cells (ECM degradation or production, apoptosis, changes in cell populations etc.) and external cues (such as light or mechanical signals, electromagnetic fields).

Recent achievements in the solid free-form fabrication of alginate-based constructs for heart valve tissue engineering illustrate the vital direction in which the cardiac regeneration techniques may develop. Duan et al. used a Fab@Home bioprinter to prepare scaffolds from gelatin/alginate -blended hydrogels^19^. Valvular interstitial cells (VICs) and aortic root sinus smooth muscle cells (SMCs) were encapsulated separately into alginate/gelatin blend before printing the heart valve root. After one week of culture, the mechanical properties of the acellular scaffolds were much lower than those of the cell-laden 3D printed hydrogels. What is now needed is further advancement in 3D deposition methods, focusing on eliciting oriented growth of the cells and the ECM, which may further enhance cardiac tissues regeneration^32,110^.

**Figure 14.**
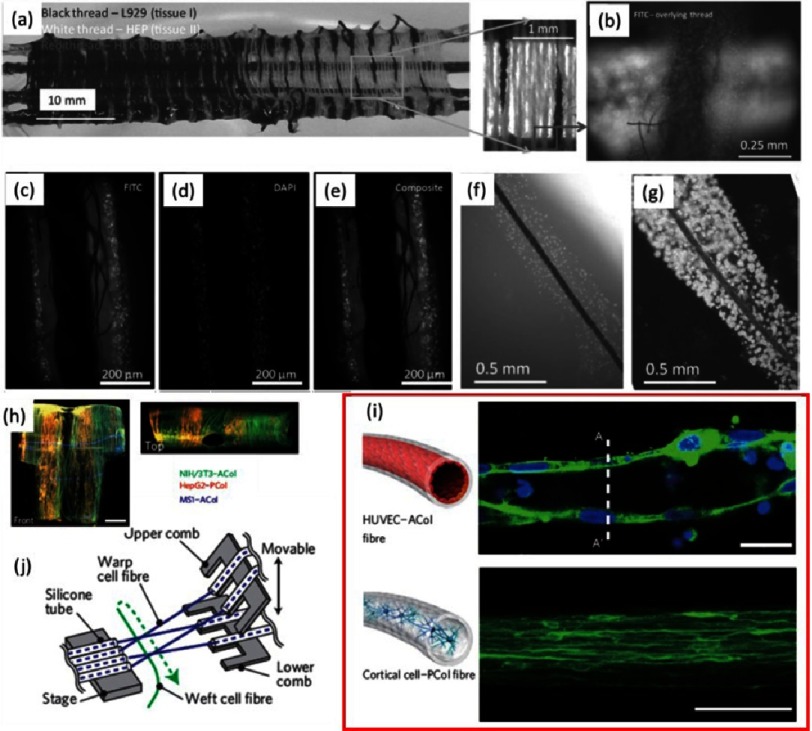
Alternative ways of 3D construct fabrication using alginate fibers. Organ weaving: connecting tissues using gradients (A). An example of manual plain weaving using three cell lines. Magnified box: basic criss-cross pattern of plain weaving. Overlaid thread containing HepG2 cells and underlying thread containing HEK-293 cells labeled CellTracker Green CMFDA (B). Fiber-supported living threads, fluorescence images of double-treated thread, including fluorescently labeled L929 cells stained with CellTracker Green CMFDA (C) and MCF-7 cells stained with nuclei stain Hoechst-33342 (D). Overlay of images (C) and (D) showing two distinguishable layers of cells (E). Living thread immediately after preparation (F). The same after 8 days of incubation (G). Pictures were reprinted with permission from (10). Fiber-based assembly of higher-order 3D macroscopic cellular structures (H). Fluorescence micrograph of a HUVEC/ACol (top panel) and primary cortical cell/PCol fiber (bottom panel) at day 35 (I). Scale bars are 20 µm. Schematic of a microfluidic weaving machine working in culture medium (J). Pictures were reprinted with permission from [11].

Although 3D printing of alginate based hydrogels is a powerful and feasible technique, effort is also needed to explore other free-form fabrication technologies. For example fibers made out of alginate could be processed using weaving, knitting and braiding (see Figure 14)^10,11^. Fibers consisting of encapsulated cells (“living” threads) are an attractive option since cells are provided directly into the dedicated location within the structure avoiding time-consuming cellular diffusion through the construct (see Figure 5A, H)^10,11^.

It is difficult to deny that replacing vital organs and tissues with fabricated composites still does not meet all the sophisticated biological and physiological requirements and reproducing their characteristics remains elusive. Many properties of alginate are not unique taken separately, but the fact that alginate combines them all makes it a valuable material that could be used to approach the challenges ahead.
